# Self-reported medication in community-dwelling older adults in Germany: results from the Berlin Initiative Study

**DOI:** 10.1186/s12877-020-1430-6

**Published:** 2020-01-21

**Authors:** Nina Mielke, Dörte Huscher, Antonios Douros, Natalie Ebert, Jens Gaedeke, Markus van der Giet, Martin K. Kuhlmann, Peter Martus, Elke Schaeffner

**Affiliations:** 1 Institute of Public Health, Charité Universitätsmedizin Berlin, Charitéplatz 1, 10117 Berlin, Germany; 2 Institute of Biometry and Clinical Epidemiology, Charité – Universitätsmedizin Berlin, Berlin, Germany; 3 Institute of Clinical Pharmacology and Toxicology, Charité – Universitätsmedizin Berlin, Berlin, Germany; 4 Department of Medicine, McGill University, Montreal, Quebec Canada; 5 Centre for Clinical Epidemiology, Lady Davis Institute, Montreal, Quebec Canada; 6 Departement of Nephrology and Medical Intensive Care, Charité – Universitätsmedizin Berlin, Berlin, Germany; 7 Department of Nephrology, Vivantes Klinikum im Friedrichshain, Berlin, Germany; 8 Institute of Clinical Epidemiology and Medical Biostatistics, Eberhard Karls-University, Tübingen, Germany

**Keywords:** Older adults, Medication, Polypharmacy, Potentially inappropriate medication, Prescription drugs, Over-the-counter drugs, Epidemiology

## Abstract

**Background:**

Older adults have the highest drug utilization due to multimorbidity. Although the number of people over age 70 is expected to double within the next decades, population-based data on their medication patterns are scarce especially in combination with polypharmacy and potentially inappropriate medication (PIM). Our objective was to analyse the frequency of polypharmacy, pattern of prescription (PD) and over-the-counter (OTC) drug usage, and PIMs according to age and gender in a population-based cohort of very old adults in Germany.

**Methods:**

Cross-sectional baseline data of the Berlin Initiative Study, a prospective cohort study of community-dwelling adults aged ≥70 years with a standardized interview including demographics, lifestyle variables, co-morbidities, and medication assessment were analysed. Medication data were coded using the Anatomical Therapeutic Chemical (ATC) classification. Age- and sex-standardized descriptive analysis of polypharmacy (≥5 drugs, PD and OTC vs. PD only and regular and on demand drugs vs regular only), medication frequency and distribution, including PIMs, was performed by age (</≥80) and gender.

**Results:**

Of 2069 participants with an average age of 79.5 years, 97% (95%CI [96%;98%]) took at least one drug and on average 6.2 drugs (SD = 3.5) with about 40 to 66% fulfilling the criteria of polypharmacy depending on the definition. Regarding drug type more female participants took a combination of PD and OTC (male: 68%, 95%CI [65%;72%]); female: 78%, 95%CI [76%;80%]). Most frequently used were drugs for cardiovascular diseases (85%, 95%CI [83%;86%]). Medication frequency increased among participants aged ≥80 years, especially for cardiovascular drugs, antithrombotics, psychoanaleptics and dietary supplements. Among the top ten prescription drugs were mainly cardiovascular drugs including lipid-lowering agents (simvastatin), beta-blockers (metoprolol, bisoprolol) and ACE inhibitors (ramipril). The most common OTC drug was acetylsalicylic acid (35%; 95%CI [33%;37%])). Dose-independent PIM were identified for 15% of the participants.

**Conclusions:**

Polypharmacy was excessive in older adults, with not only PD but also OTC drugs contributing to the high point prevalence. The medication patterns reflected the treatment of chronic diseases in this age group. There was even an increase in medication frequency between below and above 80 years especially for drugs of cardiovascular diseases, antithrombotic medication, psychoanaleptics, and dietary supplements.

## Background

Older adults often suffer from multiple morbidities whose treatment is associated with a complexity that often leads to polypharmacy [1]. The most commonly used definition of polypharmacy is the concomitant use of five or more drugs [2]. Even if this definition is applied, the reported prevalence of polypharmacy vary widely between 27% and 84% depending on age, morbidity, country, population group (primary care vs. hospitalized) and year of data collection [3, 4]. For population-based studies, prevalence of polypharmacy ranged from 40 to 67% [3, 5, 6]. Polypharmacy is associated with increased risks for adverse drug reactions leading to adverse events such as falls, hospital admissions, and mortality [7–12]. Such events are not only harmful to individuals but also increase healthcare costs considerably [13, 14]. Another aspect concerning the definition of polypharmacy is the inclusion of prescription drugs (PD) only or all medication including over-the-counter (OTC) drugs. This is often related to data availability and defining polypharmacy is often limited to prescription drugs [15, 16]. But in addition to prescription drugs, OTC drugs also contribute to the risks that are associated with polypharmacy [17]. Also related to polypharmacy are potentially inappropriate medications (PIM), where the risks of a drug are outweighed by its clinical benefit [18]. The overall PIM prevalence for community-dwelling older adults is found to be about 20% [19]. With polypharmacy, the risk for taking PIMs is increasing [20, 21].

Most of the medication is taken by older adults despite the fact that only about one-fifth of the European Union population is aged 65 and older [22]. Considering the demographic shift, the number of people above the age of 70 is predicted to double within the next decades and the proportion of the population 80 years and older is expected to be further increasing from 5% in 2010 to 15% in 2050 [23]. Furthermore, data from Italy showed that polypharmacy has increased from 43 to 53% over a period of 10 years [5]. Therefore, the burden of disease of this age group becomes even more important for the health systems [24]. Although this increasing fraction of older individuals consumes most of the medication and therefore exhibits the highest frequency of polypharmacy, little is known about the medication patterns of older adults when considering both prescription and OTC drugs. This includes the number and the type of drugs (prescription or OTC drugs) including PIMs. The German national surveys (GNHIES98, DEGS1) excluded individuals older than 79 years due to feasibility reasons [25, 26] and thus do not reflect the full range of the age-related changes due to the demographic shift. Furthermore, most other studies investigating medication patterns in Germany used dispensation data primarily from statutory health insurance companies’ providers [20, 27]. Thus, information on the presumably large part of OTC drugs cannot be derived from these data.

The aim of this study was to analyse if the frequency of polypharmacy, patterns of prescription and OTC drug usage, and PIMs differed according to age and gender in an older population using data from the Berlin Initiative Study (BIS).

## Methods

### Study population

The BIS is a prospective longitudinal population-based cohort study. Concept and design of the BIS are described in detail elsewhere [28, 29]. In brief, the BIS baseline visit was conducted from November 2009 until July 2011 and 2069 participants living in Berlin and surroundings were enrolled. Inclusion criteria were AOK membership [“Allgemeine Ortskrankenkasse” (AOK)-Nordost - Berlin’s largest statutory health insurance fund] and an age ≥ 70 years. We chose to cooperate with the AOK as this is one of the health insurance funds within Germany that covers the largest number of individuals above the age of 70 years. Dialysis patients and kidney transplant recipients, as well as nursing home residents, were excluded at baseline. An oversampling of very old age groups was carried out on purpose to be able to provide reliable numbers also for these age ranges [29]. Participants who were, for physical reasons, not able to come to a study site were visited at home. The study visit included a standardized computer-based questionnaire asking about demographics, lifestyle variables, co-morbidities, and medication as well as the assessment of anthropometric data (height, weight). Measurements were carried out according to pre-specified standardized operating procedures. Blood and urine samples were taken for instant analysis. Prior to the study, written informed consent was obtained from all participants. The study was approved by the local ethics committee, Charité, Berlin, Germany (EA2/009/08). ICD-10 (tenth Revision of the International Statistical Classification of Diseases and Related Health Problems) coded insurance claims data complemented these data.

### Medication assessment

Medication data were collected by self-report and coded using the Anatomical Therapeutic Chemical (ATC) classification system [30]. Participants were asked beforehand to bring all their medications (packages/blisters) as well as medication lists to the study visit, where a medically trained staff member conducted the medication assessment. All current regular and on-demand prescription and OTC medications were recorded, based on medication plans, medication packages, and patient self-reports, and entered into the standardized computer-based questionnaire. The questionnaire was linked to a drug database which automatically assigned relevant drug information including prescription requirement and the ATC code on entry. This could be provided for 86.3% of all drugs and for 100% of the prescription drugs. All others, mainly vitamins, minerals, and dietary supplements were coded manually. Medication was classified as either prescription drugs or OTC drugs. OTC drugs were defined as herbal medicines, dietary supplements, or nonprescription drugs according to the medicinal products act [31]. Medication frequencies at the ATC level are only shown for point prevalences higher than 5% unless it is a top 10 listing. We categorized the number of drugs utilized into groups of 0, 1–4, 5–9 and ≥ 10. Polypharmacy was defined as (1) taking 5 or more regular and on demand PD and OTC drugs, (2) 5 or more regular and on demand PD or (3) 5 and more regular PD.

### Definition and identification of PIMs

Potentially inappropriate medications for older adults (PIMs) were identified by application of the Germany-specific PRISCUS list [32]. The list was compiled through preliminary qualitative analyses of international PIM lists, systematic literature search and a panel of experts through a Delphi process. As exact dosing was not available in our dataset, we identified dose-independent PIMs based on the ATC code.

### Variables

The standardized face to face interview included (1) sociodemographic factors, (2) socioeconomic status (income and education; CASMIN [33]) (3) risk factors such as blood pressure (mean of two measurements), body mass index (BMI, </≥30), smoking (ever, never), alcohol intake (≥3 times per week, ≤2 times per week to 1 time per month, < 1 time per month), diabetes mellitus (intake of antidiabetic medication or haemoglobin A1c [HbA1c]-level of > 6.5%, yes/no), arterial hypertension (intake of antihypertensive medication, yes/no), myocardial infarction, stroke, and cancer (all self-reported, yes/no). The age-adjusted Charlson Comorbidity Index (CCI) [34, 35] was assessed by reviewing the AOK claims data. Glomerular filtration rate (GFR) was calculated by the BIS2 equation [36] based on serum creatinine and cystatin C and analysed in two categories (</≥60 ml/min/1.73m^2^).

### Statistical Analysis

The aim of the analysis was to investigate the medication frequency and distribution among a population of older adults below and above 80 years. To account for the oversampling of older age groups (≥90 years) which had been applied during enrolment, we weighted our data according to the age and sex distribution of the source population (AOK-Nordost section Berlin and surroundings). According to the available population data from the AOK, weighting by sex and age was performed individually in 5-year increments up to the age of 89 and cumulatively for ≥90 years. Weighted counts were only rounded to the nearest integer for tables. Descriptive analysis included absolute and relative frequencies, means, standard deviations, and medians, standardized by age and sex. Differences between the age groups were analysed using chi-square tests for proportions. 95%-confidence intervals (95%CI) were calculated for selected proportions. No imputation for missing values was applied with the exception of the ATC code. Formally, the level of significance was 0.05 (two-sided) for each analysis; no adjustment for multiple testing was done. Commercially available software (IBM SPSS Statistics 25.0 and R 3.4.3) was used.

## Results

### Main baseline characteristics of the study cohort by the number of drugs

Cross-sectional BIS-baseline data on medication information for analysis were available for all 2069 participants. The age and sex distribution of the subpopulation aged ≥70 years of the AOK-Nordost-Berlin was used for weighting all analyses. Table 1 displays baseline characteristics by the number of drugs. Of all study participants aged 70 to 99 years, 97% (95%CI [96%;98%]) took at least one drug; on average 6.2 drugs (SD = 3.5; median = 6.0, IQR [4.0;8.0]) were taken. Mean age was 79.5 years, and more females (63%) were participating. Approximately half of the participants described their general state of health as “good”. The unstandardized main characteristics of the study population by the number of drugs are displayed in Additional file 1: Table S1.

**Table 1:** Main baseline characteristics of the total BIS cohort by number of drugs

		Number of drugs			
	Total	0	1–4	5–9	≥10
n (%)	2069 (100)	60 (3)	635 (31)	1044 (50)	330 (16)
Age (mean ± SD), years	79.5 ± 6.6	76.4 ± 5.7	78.1 ± 6.3	80.2 ± 6.6	80.7 ± 6.6
Female, n (%)	1307 (63)	20 (34)	384 (60)	685 (66)	218 (66)
Education (CASMIN-short)^a^, n (%)					
Low	1285 (62)	36 (59)	397 (63)	648 (62)	205 (62)
Middle	434 (21)	11 (18)	118 (19)	238 (23)	67 (20)
High	340 (17)	14 (23)	118 (19)	152 (15)	57 (17)
Income, € (%)					
< 1000 €	639 (36)	17 (32)	200 (37)	321 (36)	101 (36)
1000–1999 €	1021 (58)	33 (61)	306 (57)	516 (58)	166 (60)
≥ 2000 €	106 (6)	4 (7)	35 (7)	56 (6)	11 (4)
Hypertension^b^, n (%)	1629 (79)	n.a.	396 (62)	916 (88)	317 (96)
Diabetes mellitus^c^, n (%)	538 (26)	2 (3)	84 (13)	299 (29)	153 (47)
Myocardial infarction, n (%)	247 (12)	0	32 (5)	135 (13)	80 (24)
Stroke, n (%)	172 (8)	1 (1)	35 (6)	94 (9)	42 (13)
Cancer, n (%)	435 (21)	8 (13)	118 (19)	218 (21)	92 (28)
eGFR_BIS2_^d^ < 60 ml/min/1.73m^2^, n (%)	1041 (50)	10 (17)	234 (37)	573 (55)	223 (68)
CCI (mean ± SD)	6.9 ± 3.0	4.4 ± 2.1	5.6 ± 2.4	7.2 ± 2.8	9.0 ± 3.3
BMI ≥ 30 kg/m^2^, n (%)	573 (28)	3 (5)	148 (23)	300 (29)	122 (37)
Smoking (ever), n (%)	934 (45)	36 (59)	253 (40)	486 (47)	159 (48)
Alcohol intake (%)					
< 1/month	964 (47)	19 (32)	253 (40)	505 (49)	187 (57)
≥ 1/month - 2/week	712 (35)	23 (38)	249 (40)	340 (33)	100 (31)
≥3/week – daily	376 (18)	19 (31)	127 (20)	190 (18)	40 (12)
Physical activity, n (%)					
< 1/week	528 (26)	10 (16)	96 (15)	287 (28)	135 (41)
1–5/week	947 (46)	16 (26)	310 (49)	484 (47)	138 (42)
> 5/week	588 (29)	35 (58)	229 (36)	268 (26)	57 (17)
Subjective general state of health, n (%)					
excellent	86 (4)	11 (18)	44 (7)	27 (3)	4 (1)
good	966 (47)	42 (69)	392 (62)	443 (43)	89 (27)
moderate	796 (39)	7 (12)	176 (28)	446 (43)	166 (51)
poor	180 (9)	1 (1)	17 (3)	106 (10)	57 (17)
very poor	29 (1)	0	1 (0.2)	15 (1)	12 (4)
Drug category^e^, n (%)					
OTC only	62 (3)	n.a.	58 (9)	4 (0.4)	0
PD only	407 (20)	n.a.	241 (38)	145 (14)	21 (6)
PD and OTC combined	1539 (74)	n.a.	336 (53)	895 (86)	308 (94)
PIM^f^, n (%)	311 (15)	n.a.	36 (6)	177 (17)	98 (30)

Data are means (SD, range) or absolute numbers (%).^a^CASMIN (Comparative Analysis of Social Mobility in Industrial Nations) [28]; ^b^Hypertension defined as prescription of antihypertensive medication. ^c^Diabetes defined as either HbA1c > 6.5%, or prescription of antidiabetic medication; ^d^eGFR_BIS2_ = GFR estimated by the BIS2 equation; ^e^drug category (PD – prescription drugs; OTC – over-the-counter); ^f^PIM: Potentially Inappropriate Medications – dose independent; n.a. not applicable; Data are standardized

Considering the number of drugs, 31% (95%CI [29%;33%]) took 1–4 drugs, 50% (95%CI [48%;53%]) 5–9, and 16% (95%CI [14%;18%]) ten or more drugs. With a rising number of drugs we observed an increase in age, and participants reporting higher numbers of drugs exhibited higher frequencies of morbidities such as hypertension, diabetes, myocardial infarction, stroke, cancer, reduced kidney function, and obesity. This also held true for the Charlson Comorbidity Index (CCI). Differentiating between the drug categories shows that most participants (74%, 95%CI [72%;76%]) used a combination of OTCs and PDs. About half (53%; 95%CI [49%;57%]) of the participants with 1–4 drugs, 86% (95%CI [83%;88%]) with 5–9 drugs and 94% (95%CI [90%;96%]) of the participants with ten or more drugs were taking a combination of OTCs and prescription medication. One-fifth of the participants were taking PDs only and 3% OTCs only. About one out of seven participants were prescribed at least one drug classified as a dose-independent PIM for older people. The frequency of PIMs increased from 6% (95%CI [4%;8%]) in participants with 1–4 drugs to 30% (95%CI [25%;35%]) in the ones with ten or more drugs.

### Medication distribution by age and gender

**Fig. 1:**
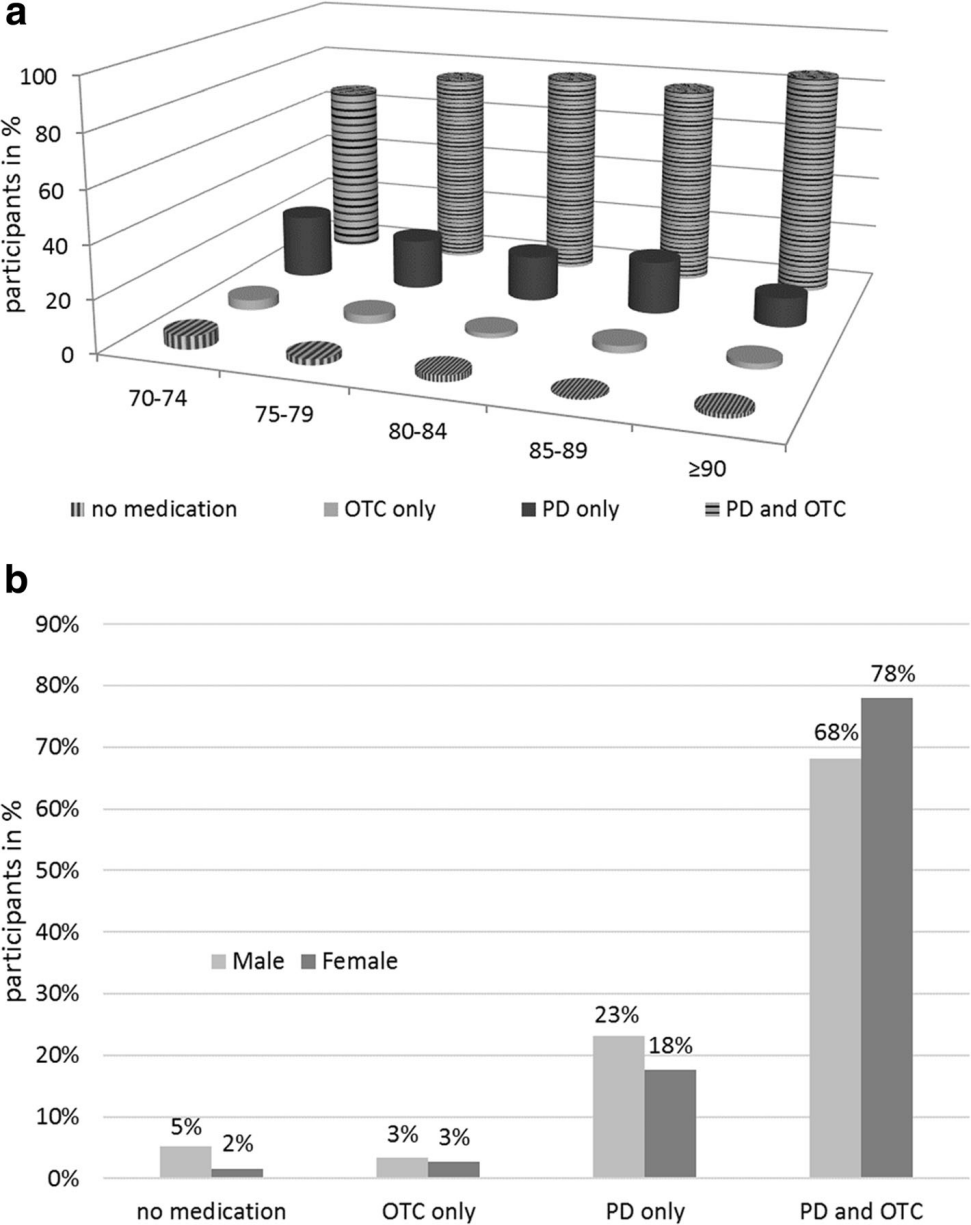
Medication categories by age (**a**) and gender (**b**). Data are presented on individual participant level; OTC (over-the-counter medication), PD (prescription medication)

For the analyses by age and gender participants were classified into the following four categories: no medication (1), OTC medication only (2), prescription medication only (3) and prescription as well as OTC drugs (4). Age and gender-specific analyses (Fig. 1) showed little difference in the number of participants using no medication or OTC only. The frequency of participants taking only prescription medication decreased from 24% (95%CI [21%;28%]) to 11% (95%CI [7%;17%]) over age groups and increased from 67% (95%CI [63%;70%]) in the youngest to 85% (95%CI [80%;90%]) in the oldest for the ones who took both, prescription and OTC drugs. 23% (95%CI [20%;26%]) of males took prescription drugs only and 68% (95%CI [65%;72%]) both prescription and OTC drugs as compared to 18% (95%CI [16%;20%]) and 78% (95%CI [76%;80%]) in females, respectively.

### Polypharmacy

The number of drugs taken stratified by age groups is summarized in Table 2. There were nearly twice as many participants taking five or more drugs compared to those taking four or fewer drugs. Across all age groups, more than half of the participants fell into the category polypharmacy (≥5 drugs). The number of participants with polypharmacy was steadily increasing over the age groups from 54% (95%CI [50%;58%]) in the youngest age group up to 76% (95%CI [70%;83%]) in the ≥90-year-olds. Applying the strict definition of polypharmacy (≥5 regularly taken prescription drugs) except for the ages 70–74, more than 40% of the participants were affected.

**Table 2:** Number of drugs taken by age groups

		Age groups				
	Total	70–74	75–79	80–84	85–89	≥90
n	2069	644 (31)	541 (26)	426 (21)	273 (13)	185 (9)
Number of drugs (mean ± SD)	6.2 ± 3.5	5.4 ± 3.5	6.3 ± 3.4	6.8 ± 3.5	6.7 ± 3.1	7.1 ± 3.4
Number of drugs [median (IQR)]	6 (4;8)	5 (3;7)	6 (4;8)	7 (4;9)	6 (4;8)	7 (5;9)
Number of drugs [n (%)]						
0	60 (3)	33 (5)	13 (2)	11 (3)	1 (1)	3 (2)
1–4	635 (31)	264 (41)	161 (30)	98 (23)	73 (27)	40 (22)
5–9	1044 (50)	273 (42)	283 (52)	230 (54)	153 (56)	104 (56)
> =10	330 (16)	75 (12)	84 (16)	87 (21)	46 (17)	37 (20)
OTC						
0	468 (23)	189 (29)	115 (21)	84 (20)	56 (20)	24 (13)
1–4	1491 (72)	433 (67)	402 (74)	312 (73)	197 (72)	147 (79)
> =5	110 (5)	22 (3)	23 (4)	30 (7)	20 (7)	15 (8)
PD						
0	123 (6)	58 (9)	31 (6)	18 (4)	9 (3)	7 (4)
1–4	1022 (49)	359 (56)	263 (49)	189 (44)	131 (48)	80 (43)
> =5	924 (45)	227 (35)	247 (46)	218 (51)	133 (49)	98 (53)
Polypharmacy only regular PDs	829 (40)	204 (32)	223 (41)	198 (47)	122 (45)	81 (44)

Data are standardized

### Top ten ATC anatomical main groups and their subgroups

Of the top ten anatomical main groups (level of indication), drugs for the treatment of the cardiovascular system (ATC code C) were most commonly used (85%, 95%CI [83%;86%]). Of those, agents acting on the renin-angiotensin system (C09) were the most frequent, followed by beta blockers (C07) (Table 3). The second most common main group was drugs of the alimentary tract and metabolism (A; 64%, 95%CI [62%;66%]), where mineral supplements (A12) and drugs for acid-related disorders (A02) were the most frequent subgroups. Medications for the treatment of blood and blood-forming organs (B) came third (49%, 95%CI [47%;51%]) with antithrombotic agents (B01) as the main subgroup (details Additional file 2: Table S2). These were followed by medication for treatment of the nervous system (N; 38%, 95%CI [36%;40%]) and musculoskeletal system (M; 34%, 95%CI [32%;36%]) with analgesics (N02) as well as anti-inflammatory and antirheumatic drugs (M01) as most frequently used subgroups.

**Table 3:** The ten most frequent (prescription drugs and over-the-counter medication) anatomical main groups (bold) and their most frequent therapeutic subgroups stratified by age [n (%)]

	total *N* = 2069	70–79 *N* = 1186	≥80 *N* = 883	*p*-value
**ATC anatomical main group **and subgroup				
**cardiovascular system (C)**	1748 (85)	954 (80)	795 (90)	< 0.001
agents acting on the renin-angiotensin system (C09)	1283 (62)	690 (58)	593 (67)	< 0.001
beta blocking agents (C07)	951 (46)	514 (43)	437 (50)	0.006
lipid modifying agents (C10)	756 (37)	431 (36)	325 (37)	0.84
diuretics (C03)	569 (28)	258 (22)	311 (35)	< 0.001
calcium channel blockers (C08)	525 (25)	281 (24)	243 (28)	0.050
cardiac therapy (C01)	398 (19)	155 (13)	243 (28)	< 0.001
**alimentary tract and metabolism (A)**	1329 (64)	743 (63)	586 (66)	0.090
mineral supplements (A12)	677 (33)	360 (30)	317 (36)	0.009
drugs for acid related disorders (A02)	459 (22)	257 (22)	202 (23)	0.54
drugs used in diabetes (A10)	409 (20)	251 (21)	158 (18)	0.061
vitamins (A11)	250 (12)	129 (11)	122 (14)	0.043
**blood and blood forming organs (B)**	1013 (49)	507 (43)	506 (57)	< 0.001
antithrombotic agents (B01)	949 (46)	470 (40)	480 (54)	< 0.001
antianemic preparations (B03)	123 (6)	61 (5)	61 (7)	0.094
**nervous system (N)**	783 (38)	388 (33)	396 (45)	< 0.001
analgesics (N02)	364 (18)	170 (14)	194 (22)	< 0.001
psychoanaleptics (N06)	277 (13)	119 (10)	157 (18)	< 0.001
psycholeptics (N05)	192 (9)	102 (9)	90 (10)	0.22
**musculo-skeletal system (M)**	704 (34)	402 (34)	302 (34)	0.89
antiinflammatory and antirheumatic products (M01)	386 (19)	237 (20)	150 (17)	0.080
antigout preparations (M04)	202 (10)	112 (10)	90 (10)	0.60
drugs for treatment of bone diseases (M05)	127 (6)	55 (5)	72 (8)	0.001
**systemic hormonal preparations, excl. Sex hormones and insulins (H)**	503 (24)	275 (23)	228 (26)	0.17
thyroid therapy (H03)	453 (22)	253 (21)	200 (23)	0.50
**genitourinary system and sex hormones (G)**	338 (16)	203 (17)	135(15)	0.28
urologicals (G04)	286 (14)	169 (14)	118 (13)	0.55
**various (V)**	307 (15)	152 (13)	155 (18)	0.011
general nutrients (V06)	261 (13)	126 (11)	135 (15)	0.001
**respiratory system (R)**	289 (14)	172 (15)	117 (13)	0.41
drugs for obstructive airway diseases (R03)	219 (11)	131 (11)	88 (10)	0.41
**sensory organs (S)**	154 (8)	87 (7)	67 (8)	0.85
ophthalmologicals (S01)	154 (8)	87 (7)	67 (8)	0.85

*P*-values shown are from age-group comparison by Chi^2^ test; Data are standardized

Comparing the younger (70–79 years) to the older (≥80 years) participants, there was a significant increase in the frequency of ATC main anatomical groups C, B, N and V (Table 3). Most of the drugs on therapeutic subgroup level for the treatment of the cardiovascular system (C) were significantly more often used in the 80 plus stratum. The same held true for antithrombotic agents (B01), analgesics (N02), psychoanaleptics (N06), drugs for treatment of bone disease (M05) and general nutrients (V06).

### ATC code on chemical substance level

Next, we analysed the medication on the chemical substance level. The ten most common prescription drugs included a lipid-lowering agent (simvastatin), beta-blockers (metoprolol, bisoprolol), an agent for thyroid hormone deficiency (levothyroxine sodium), medication for the treatment of cardiovascular diseases (blood pressure, heart insufficiency and heart failure: amlodipine, torasemide, ramipril), a drug for diabetes treatment (metformin) and a proton-pump inhibitor (omeprazole) (Table 4). Among the ten most frequently consumed OTCs were acetylsalicylic acid, minerals (magnesium, calcium), dietary supplements, and analgesics.

**Table 4:** Top ten most frequently used prescription medication and OTC including herbal products and supplements on ATC chemical substance level [n (%)]

	total *N* = 2069	70–79 *N* = 1186	≥80 *N* = 883	*p*-value
Prescription medication				
simvastatin	577 (28)	329 (28)	248 (28)	0.87
metoprolol	440 (21)	231 (20)	209 (24)	0.021
levothyroxine sodium	386 (19)	213 (18)	173 (20)	0.35
amlodipine	281 (14)	164 (14)	118 (13)	0.75
bisoprolol	274 (13)	150 (13)	124 (14)	0.35
torasemide	253 (12)	105 (9)	147 (17)	< 0.001
ramipril and hydrochlorothiazid	218 (11)	130 (11)	88 (10)	0.46
ramipril	216 (10)	119 (10)	97 (11)	0.48
metformin	213 (10)	144 (12)	69 (7)	0.001
omeprazole	208 (10)	115 (10)	93 (11)	0.54
OTC including herbal products and supplements				
acetylsalicylic acid	731 (35)	365 (31)	366 (42)	< 0.001
magnesium^a^	344 (17)	189 (16)	155 (18)	0.060
dietary supplement	226 (11)	109 (9)	116 (13)	0.004
calcium	160 (8)	85 (7)	75 (9)	0.068
ibuprofen	145 (7)	95 (8)	50 (6)	0.039
Ginkgo biloba	136 (7)	51 (4)	84 (10)	< 0.001
omega-3-triglycerides incl. other esters and acids	65 (3)	38 (3)	27 (3)	0.85
tocopherol (vit E)	52 (3)	31 (3)	20 (2)	0.61
paracetamol	52 (3)	27 (2)	25 (3)	0.43
Hawthorn flower	50 (2)	14 (1)	36 (4)	< 0.001

*P*-values shown are from age-group comparison by Chi^2^ test; Data are standardized

^a^magnesium (ATC: A12CC w/o A12CC30 and A12CC50)

Overall, the three most frequent drugs were acetylsalicylic acid (35%, 95%CI [33%;37%], of those 96% as antithrombotic treatment), simvastatin (28%, 95%CI [26%;30%]) and metoprolol (21%, 95%CI [20%;23%]). Age-specific analyses demonstrated a significantly higher consumption of acetylsalicylic acid and metoprolol in participants aged ≥80 as compared to < 80 years.

### Frequency of PIMs by age and gender

15% (95%CI [14%;17%]) of the participants were identified to have at least one dose-independent PIM (Table 5) in their medication. Of the participants with PIMs, 85% (95%CI [80%;88%]) had one PIM, 14% (95%CI [10%;19%]) two and 1% (95%CI [0.3%;3%]) were identified with three different PIMs. Analysis of PIMs by age and gender demonstrated only marginal differences in the prescription of PIMs on subgroup level. Out of 16 different ATC subgroups found in PIMs, psychoanaleptics (N06; 20%, 95%CI [16%;25%]), psycholeptics (N05; 19%, 95%CI [15%;24%]) and antihypertensives (C02; 14%, 95%CI [10%;19%]) were the three most common ones. The ATC subgroup classes showed no significant differences between age groups and gender. Exceptions were drugs for obstructive airway diseases (*p* = 0.049) which were found almost twice as often in younger than older participants, and psycholeptics (*p* = 0.039) which were found approximately twice as often in females.

**Table 5:** Dose-independent Potentially Inappropriate Medications (PIMs) by age and gender [n (%)]

	total *N* = 2069	70–79 *N* = 1186	≥80 *N* = 883	*p*-value
PIMs total	311 (15)	173 (15)	139 (16)	0.47
PIM: ATC subgroup (*n* = 311)				
psychoanaleptics (N06)	63 (20)	37 (22)	26 (19)	0.54
psycholeptics (N05)	59 (19)	30 (17)	30 (21)	0.35
antihypertensives (C02)	44 (14)	22 (13)	22 (16)	0.42
calcium channel blockers (C08)	34 (11)	16 (9)	18 (13)	0.30
drugs for obstructive airway diseases (R03)	33 (10)	23 (13)	9 (7)	0.049
urologicals (G04)	30 (10)	16 (9)	14 (10)	0.81
antiinflammatory and antirheumatic products (M01)	24 (8)	14 (8)	10 (7)	0.77
cardiac therapy (C01)	20 (7)	11 (6)	9 (7)	0.95
	total *N* = 2069	male *N* = 762	female *N* = 1307	*p*-value
PIMs total	311 (15)	104 (14)	208 (16)	0.17
PIM: ATC subgroup (*n* = 311)				
psychoanaleptics (N06)	63 (20)	19 (18)	44 (21)	0.55
psycholeptics (N05)	59 (19)	13 (12)	46 (22)	0.039
antihypertensives (C02)	44 (14)	20 (20)	24 (11)	0.061
calcium channel blockers (C08)	34 (11)	12 (12)	21 (10)	0.69
drugs for obstructive airway diseases (R03)	33 (10)	11 (10)	22 (11)	1.0
urologicals (G04)	30 (10)	9 (9)	20 (10)	0.79
antiinflammatory and antirheumatic products (M01)	24 (8)	9 (9)	15 (7)	0.65
cardiac therapy (C01)	20 (7)	7 (7)	13 (6)	0.85

*P*-values shown are from age-group comparison by Chi^2^ test; Data are standardized

## Discussion

This study used data of the BIS, a population-based cohort of old and very old adults [28] to give an in-depth picture of the medication pattern in a German cohort of people aged 70 and older. After standardization for the age and sex distribution of the AOK-Nordost-Berlin, our data showed that only a very small fraction of older adults were free of any medication use, whereas the majority consumed both, prescription and OTC medication with an average of 6.2 drugs. The number of drugs increased with age. Applying the widely accepted definition of polypharmacy as intake of a minimum of five regularly taken prescription drugs, about 40% of the participants met this definition, but when also considering OTC intake, this number increased to about two thirds. The analysis by drug type (OTC only, PD only, or both) showed that women took more often a combination of PDs and OTCs. The most common ATC anatomical main group was the group of drugs acting on the cardiovascular system. Medication frequency increased significantly from participants aged 70–79 years to participants 80 years and older especially for drugs for the treatment of cardiovascular diseases (e.g. beta blocker, diuretics, ACE inhibitors), antithrombotic medication, psychoanaleptics (e.g. antidepressants) and dietary supplements. On chemical substance level, simvastatin was the most commonly prescribed drug and acetylsalicylic acid the most commonly consumed OTC. Using the German-specific PRISCUS list, we identified dose-independent PIMs for 15% of the participants.

Studies that display the entire medication utilization spectrum in old age are rare. Several studies in old age investigate polypharmacy and its implications. Other studies focus on PIMs in old age. Many of these analyses use health insurance claims data, which only include information about prescribed drugs. The patient’s adherence to prescribed medications is critical to what is consumed [37]. Therefore, prescribed medications do not necessarily reflect the drugs taken regularly. In the BIS, using the approach of self-reported medication, we expect that we do not only record the prescribed medications but the medications actually taken. Again, other studies focus on OTC medication or herbal and dietary supplements. Studies such as the BIS that combine data on both drug categories with detailed phenotyping in old age are scarce and are conducted in younger populations (median age 49 years) or different health-care systems (England and Wales, Brazil) [38–40]. Thus comparison of results is difficult due to other classifications of prescription drugs and other medication patterns among adults below the age of 70 years. However, when comparing our results of the septuagenarians to the oldest age stratum (70–79 years) of the surveys of the German federal health reporting system (DEGS, GNHIES98) the high medication frequency lies within a very similar range [25, 26]. However, all other information concerning the medication spectrum is presented for the entire adult age range (18–79 years) there, so that further comparisons are not possible.

As multimorbidity increases in old age older adults fulfil the criteria of polypharmacy. Prevalence estimates of polypharmacy vary, often due to differing definitions of the number of medications that constitute polypharmacy, and diverging time periods of assessment [41]. Interestingly, general practitioners usually underrate the number of prescribed and OTC drugs especially for multiple medication users [42]. We applied different definitions of polypharmacy defined as a minimum of five drugs: regular and on demand PD and OTC, both separated and the most narrow definition including only regular PD. Independent of the prescription or OTC status, the elderly are at higher risk for adverse drug reactions [43]. Polypharmacy itself has been associated with adverse events (mortality, falls, and drug reactions), hospital admissions, increased length of stay and readmission to a hospital soon after discharge [7, 9, 16, 44]. However, not only the number of drugs varies between the definitions for polypharmacy but also the type of drugs included (prescription drugs only vs. all drugs). We decided to include all medications since it has been shown that about one-fifth of the OTC users were identified with at least one drug-related problem [17], and that at least half of the major drug-drug interactions involve OTC drugs [45]. Our data demonstrate that the number of OTCs taken by the elderly is even further increasing with age. There is a difference of 26% between the prevalence of polypharmacy only including five or more regular drugs and including five or more regular and on demand PD and OTC drugs. Thus, combining prescription and nonprescription medication as well as intermittently used drugs is important to show the full burden of polypharmacy.

The most commonly used drugs belong to the ATC main group of cardiovascular drugs. This is in accordance with other studies [38, 40, 46] and mirrors the treatment against the primary reason for death from chronic cardiovascular diseases [47]. Besides drugs for the treatment of cardiovascular diseases, the most commonly reported drugs were antithrombotic agents, drugs for the treatment of the alimentary tract and metabolism including mineral supplements, drugs for acid-related disorders, and drugs used in diabetes. Swedish studies presented similar results in their oldest age strata [48, 49]. Despite differences in health-care systems and medication assessment, the number one drug taken by the elderly is often acetylsalicylic acid [4, 46, 50–52]. In the BIS 20% of the participants used acetylsalicylic acid for primary prevention of cardiovascular events even though the clinical benefit for acetylsalicylic acid in primary prevention for older people is controversial [53–56]. In addition, among the top 10 list of OTCs were vitamins, minerals and dietary supplements for which, again, the evidence for a clinical benefit is lacking whereas the cost of these products is immense [57].

Criteria have been developed by expert panels to increase the quality in prescribing practices and medication use in older adults in order to identify and avoid PIMs for older adults [58]. The PRISCUS list was specially designed for the German pharmaceutical market [32]. In contrast to other studies on PIMs in old age, our data show lower PIM frequencies (15% vs. 25%). This could be partly due to differences in data collection. Whereas in the BIS information on medication is documented as a point prevalence, in most other studies the PIM prevalence is analysed within claims data reporting a one-year prevalence [20, 27, 59]. Secondly, regional differences in PIM prevalence have shown that there is a geographical gradient in PIM prevalence with 24/25% in western to 17/19% in eastern Germany [60]. Thirdly, the fact that we only assessed dose-independent PIMs may have caused underestimation of the prevalence in the BIS. As shown for the European region, the most frequently used PIMs belonged to the group of anxiolytics, antidepressants, and non-steroidal anti-inflammatory drugs [19]. In the BIS, we identified in addition the PIMs nifedipine and doxazosin in similarly frequent amounts. This is in accordance with data from Germany during the same period [20, 27], which have also shown that women receive PIMs more often than men [20, 27, 59]. We could not find these gender differences in our study maybe due to the relatively small total number of PIMs.

The strengths of our study are its population-based approach and in contrast to most other studies its reasonable sample size of 2069 old and very old adults with an age range of 70–99 years. As the original study population was by (oversampling) design older than the respective source, we used age and sex specific weights in order to base our analysis on an age-sex-ratio of the source population during the same time period. Furthermore, the thorough (including medication plans and packaging) assessment of all medication taking into account both, prescription and OTC drugs, and the detailed phenotyping of the participants contributes to the strength of the study.

Some limitations also deserve mention. Firstly, the assessment of the medication was self-reported supported by medication plans if available. We were not able to assess pill counts or dosing which would have added more detail to the burden of medication in old age. Secondly, we were unable to verify if the participants had in fact consumed the medication. Thirdly, the medication assessment of 13.7% of the drugs could not deliver an ATC code information automatically. In such situations, all available medication information systems were accessed. In case a drug could still not be identified, an ATC code was issued on an aggregated level. This was mainly the case for vitamins, minerals or dietary supplements (A11, A12 and V06). Thus, the percentage of non-classified drugs in terms of ATC code was reduced although a misclassification bias cannot be completely excluded. Fourthly, the BIS is a local cohort placed in Berlin with participants of a large health insurance company, AOK. Despite standardizations for the age and sex distribution of the source population statements beyond age and sex differences, e.g. differences between urban and rural areas are not covered with this standardization and can thus not be ruled out.

## Conclusion

About two-thirds of old and very old adults met the definition of polypharmacy and thus face its associated risks. Polypharmacy was composed of both, prescription and over the counter drugs. Using a stricter definition of polypharmacy (five or more regular PD) reduces the prevalence potentially underestimating its burden.

The medication pattern reflected the treatment of chronic diseases in this age group. The increase in medication frequency between septuagenarians and the ones 80 years and older especially for cardiovascular disease drugs, antithrombotic medication, psychoanaleptics, and dietary supplements, demonstrates the importance of subdividing the upper age group when analysing medication patterns in older adults.

Future research should capture the indications for medication intake in order to be able to differentiate between primary or secondary prevention. This may make it easier to identify approaches to reduce the number of drugs especially in multimorbid older adults.

## Supplementary information

**Additional file 1: Table S1.** Unstandardized main baseline characteristics of the total BIS cohort by number of drugs.

**Additional file 2: Table S2.** Antithrombotic agents and their chemical subgroups stratified by age [n (%)].

## Data Availability

The datasets used and analysed during the current study are available from the corresponding author on reasonable request.

## Abbreviations

ACE: Angiotensin-converting enzyme
AOK: Berlin’s largest statutory health insurance fund (*Allgemeine Ortskrankenkasse*)
ATC: Anatomical Therapeutic Chemical
BIS: Berlin Initiative Study
BMI: Body mass index
CCI: Charlson Comorbidity Index
DEGS1: German Health Interview and Examination Survey for Adults (*Studie zur Gesundheit Erwachsener in Deutschland*)
GFR: Glomerular filtration rate
GNHIES98: German National Health Interview and Examination Survey 1998
HbA1c: Haemoglobin A1c
ICD-10: Tenth Revision of the International Statistical Classification of Diseases and Related Health Problems
OTC: Over-the-counter
PD: Prescription drug
PIM: Potentially inappropriate medication
