# Supplementary material for: Self-reported medication in community-dwelling older adults in Germany: results from the Berlin Initiative Study
Source: BMC Geriatr. 2020 Jan 21;20:22. doi: 10.1186/s12877-020-1430-6 (PMC6974973; doi:10.1186/s12877-020-1430-6)
Supplement: Supplementary file 1 — Additional file 1: Table S1. Unstandardized main baseline characteristics of the total BIS cohort by number of drugs. [file 12877_2020_1430_MOESM1_ESM.docx]

**Table 1: Unstandardized main baseline** **characteristics of the total BIS cohort by number of drugs**.

|  |  | Number of drugs | | | |
| --- | --- | --- | --- | --- | --- |
|  | Total | 0 | 1-4 | 5-9 | >=10 |
| n (%) | 2069 (100) | 60 (3) | 629 (30) | 1045 (51) | 335 (16) |
| Age (mean±SD), years | 80.4±6.7 | 77.7±6.6 | 79.1±6.8 | 80.9±6.6 | 81.4±6.6 |
| Female, n (%) | 1088 (53) | 17 (28) | 330 (53) | 564 (54) | 177 (53) |
| Education  (CASMIN-short)^1^,  n (%) |  |  |  |  |  |
| Low | 1244 (60) | 34 (57) | 377 (60) | 636 (61) | 197 (59) |
| Middle | 410 (20) | 9 (15) | 114 (18) | 223 (21) | 64 (19) |
| High | 405 (20) | 17 (28) | 135 (22) | 181 (17) | 72 (22) |
| Income, € (%) |  |  |  |  |  |
| <1000 € | 574 (33) | 16 (30) | 182 (34) | 286 (32) | 90 (32) |
| 1000-1999 € | 1071 (61) | 34 (64) | 316 (59) | 543 (61) | 178 (63) |
| ≥2000 € | 117 (7) | 3 (6) | 38 (7) | 60 (7) | 16 (6) |
| Hypertension^2^, n (%) | 1634 (79) | 0 | 391 (62) | 919 (88) | 324 (97) |
| Diabetes mellitus^3^, n (%) | 539 (26) | 2 (3) | 85 (14) | 302 (29) | 150 (45) |
| Myocardial Infarction, n (%) | 286 (14) | 0 | 38 (6) | 161 (16) | 87 (26) |
| Stroke, n (%) | 177 (9) | 1 (2) | 34 (6) | 93 (9) | 49 (15) |
| Cancer, n (%) | 465 (23) | 9 (15) | 124 (20) | 238 (23) | 94 (28) |
| eGFR_BIS2_^4^ < 60 ml/min/1.73m^2^ , n (%) | 1096 (53) | 13 (22) | 251 (40) | 601 (58) | 231 (69) |
| CCI (mean±SD) | 7.2±3.1 | 4.8±2.4 | 5.8±2.5 | 7.4±2.9 | 9.4±3.4 |
| BMI ≥ 30 kg/m^2^, n (%) | 546 (26) | 3 (5) | 136 (22) | 291 (28) | 116 (35) |
| Smoking (ever), n (%) | 1029 (50) | 34 (57) | 272 (43) | 543 (52) | 180 (54) |
| Alcohol intake (%) |  |  |  |  |  |
| <1/month | 914 (45) | 20 (33) | 241 (39) | 475 (46) | 178 (54) |
| ≥1/month - 2/week | 724 (35) | 22 (37) | 247 (40) | 346 (33) | 109 (33) |
| ≥3/week – daily | 414 (20) | 18 (30) | 135 (22) | 215 (21) | 46 (14) |
| Physical activity, n (%) |  |  |  |  |  |
| <1/week | 527 (26) | 10 (17) | 99 (16) | 284 (27) | 134 (40) |
| 1-5/week | 961 (47) | 17 (28) | 312 (50) | 488 (47) | 144 (43) |
| >5/week | 576 (28) | 33 (55) | 217 (35) | 269 (26) | 57 (17) |
| Subjective general state of health, n (%) |  |  |  |  |  |
| excellent | 88 (4) | 10 (17) | 47 (8) | 26 (3) | 5 (2) |
| good | 977 (48) | 41 (68) | 390 (63) | 455 (44) | 91 (27) |
| moderate | 782 (38) | 7 (12) | 168 (27) | 442 (43) | 165 (50) |
| poor | 179 (9) | 2 (3) | 18 (3) | 99 (10) | 60 (18) |
| very poor | 28 (1) | 0 | 1 (0.2) | 15 (1) | 12 (4) |
| Drug category^5^, n (%) | | | | | |
| OTC only | 61 (3) | n.a. | 57 (9) | 4 (1) | 0 |
| PD only | 406 (20) | n.a. | 238 (38) | 148 (14) | 20 (6) |
| PD and OTC combined | 1542 (75) | n.a. | 334 (53) | 893 (86) | 315 (94) |
| PIM^6^, n (%) | 314 (15) | n.a. | 37 (6) | 176 (17) | 101 (30) |
|  |  |  |  |  |  |

Data are means (SD, range) or absolute numbers (%).^1^CASMIN (Comparative Analysis of Social Mobility in Industrial Nations) [28]; ^2^Hypertension defined as prescription of antihypertensive medication. ^3^Diabetes defined as either HbA1c >6.5%, or prescription of antidiabetic medication; ^4^eGFR_BIS2_ = GFR estimated by the BIS2 equation; ^5^drug category (PD – prescription drugs; OTC – over-the-counter); ^6^PIM: Potentially Inappropriate Medications – dose independent; n.a. not applicable
