# Supplementary material for: Self-reported medication in community-dwelling older adults in Germany: results from the Berlin Initiative Study
Source: BMC Geriatr. 2020 Jan 21;20:22. doi: 10.1186/s12877-020-1430-6 (PMC6974973; doi:10.1186/s12877-020-1430-6)
Supplement: Supplementary file 2 — Additional file 2: Table S2. Antithrombotic agents and their chemical subgroups stratified by age [n (%)]. [file 12877_2020_1430_MOESM2_ESM.docx]

**Supplement Table 2: Antithrombotic agents and their chemical subgroups stratified by age [n (%)].**

|  | **total**  **N=2069** | **70-79**  **N=1186** | **≥80**  **N=883** | **p-value** |
| --- | --- | --- | --- | --- |
| antithrombotic agents (B01) | 949 (46) | 470 (40) | 480 (54) | <0.001 |
| vitamin K antagonists (B01AA) | 197 (10) | 98 (8) | 99 (11) | 0.024 |
| heparins (B01AB) | 4 (0.2) | 3 (0.3) | 0 | 0.14 |
| platelet aggregation inhibitors excl. heparin (B01AC) | 778 (38) | 388 (33) | 389 (44) | <0.001 |
| enzymes (B01AD) | 0 | 0 | 0 |  |
| direct thrombin inhibitors (B01AE) | 0 | 0 | 0 |  |
| direct factor Xa inhibitors (B01AF) | 0 | 0 | 0 |  |
| other antithrombotic agents (B01AX) | 0 | 0 | 0 |  |

P-values shown are from age-group comparison by Chi^2^ test; Data are standardized
